# Various Solvent Systems for Solubility Enhancement of Enrofloxacin

**DOI:** 10.4103/0250-474X.51958

**Published:** 2009

**Authors:** Neelam Seedher, Pooja Agarwal

**Affiliations:** Department of Chemistry, Panjab University, Chandigarh-160 014, India

**Keywords:** Enrofloxacin, solubility, co-solvents, surfactants, sodium dodecylsulphate

## Abstract

Solubility enhancement of antimicrobial drug enrofloxacin has been studied using a series of co-solvents and surfactants. Aqueous solubility of enrofloxacin could be increased up to 26 times. Co-solvents alone produced only small increase in solubility. However, the combined effect of co-solvents and buffer was synergistic and a large increase in solubility could be attained. Ionic surfactants were found to be much better solubilizing agents than non-ionic surfactant. Amongst ionic surfactants, solubility was found to be very high in anionic surfactant, sodium dodecylsulphate as compared to the cationic surfactant, cetyltrimethylammonium bromide. Up to 3.8 mg/ml of enrofloxacin could be dissolved in sodium dodecylsulphate. Mechanism of solubilization has been proposed and surfactant solubilization parameters have been calculated.

The development of fluoroquinolone class of antibiotics has been a major breakthrough in the treatment of bacterial infections. These medications are active against many bacterial types including pseudomonas and are not associated with serious side effects that plagued the aminoglycoside group. Enrofloxacin, a broad spectrum fluoroquinolone antibiotic, which has shown efficacy for veterinary use[1]. Some physico-chemical properties of enrofloxacin have been reported[2].

Enrofloxacin is beset with the disadvantage of poor aqueous solubility. The very poor aqueous solubility and wettability of the drug gives rise to difficulties in the design of pharmaceutical formulations and leads to variable bioavailability. The pH-solubility profile of enrofloxacin has been reported by Lizondo *et al*.[2]. However, there appear to be no reports on the enhancement of solubility of enrofloxacin. The use of co-solvents is a highly effective technique to enhance the solubility of poorly-soluble drugs[3–5]. In the present study an attempt has been made to increase the solubility of enrofloxacin using a series of co-solvents and surfactants.

Pure enrofloxacin was obtained as a gift sample from Ranbaxy Laboratories Ltd. India. All solvents used were of analytical grade. Water used was double distilled in all glass apparatus. Ultraviolet absorption spectrophotometric technique was employed for the estimation of enrofloxacin. Due to limited solubility of enrofloxacin in water/buffer, 0.02 N sodium hydroxide was used as solvent. Extinction coefficient of drug, determined at 271 nm, was used to estimate the unknown drug concentration.

For the determination of solubility, excess of drug was placed in contact with 5 ml of solvent in sealed conical flasks. The flasks were maintained at 25° and the contents were stirred on a magnetic stirrer for the required time period (3½/24 h). The solution was centrifuged and the supernatant was filtered through 0.45 μm filter. The absorbance of clear solution was determined at λ_max_ of the drug after suitable dilution with the appropriate solvent. The concentration of drug was determined from Beer Lambert law using extinction coefficients, determined in the relevant solvent. The solubility was calculated by multiplying the drug concentration, so obtained, by the appropriate dilution factor. The reported data are an average of three determinations.

The solubility of enrofloxacin in water, 0.1 M phosphate buffer (PB, pH 7.4), 10-40% concentration of co-solvents: polyethylene glycol 400 (PEG 400), propylene glycol (PG), glycerol and ethanol, 25 and 50 mM micellar concentrations (total surfactant concentration; CMC) of a cationic (cetyl trimethylammonium bromide, CTAB), anionic (sodium dodecylsulfate, SDS) and non-ionic (polyoxyethylene (20) sorbitan monooleate, Tween 80) surfactant and 1:1 and 2:1 PEG 8000:drug solid dispersions, prepared by solvent evaporation method[6], was determined at 25°. Water as well as PB (pH 7.4) medium was used for solubility determination in each case.

Enrofloxacin is an amphoteric drug with pK_a1_ = 5.94, corresponding to carboxyl group and pK_a2_ = 8.70, corresponding to basic piperazinyl group and the isoelectric pH= 7.32. The lipophilicity, measured as octanol-aqueous buffer partition coefficient (log D) is 3.48 at pH 7.00 pH 7.002[2]. Thus at near neutral pH drug exists in zwitterionic form and has high lipohilicity. Aqueous solubility of enrofloxacin in water at 25° was found to be 146 μg/ml and therefore, the drug can be categorized as poorly soluble. The solubility was seen to increase from 146 to 182 μg/ml by using 0.1 M PB (pH 7.4) as solvent.

The use of co-solvents is a simple and effective technique, widely used to enhance the solubility of poorly soluble drugs[3–5]. Initially the time period for drug dissolution was kept 3½ h (210 min) for all the solvents. The amount of drug dissolved and the initial dissolution rate in water, phosphate buffer and 10-40% concentration of co-solvents in water as well as 0.1 M PB (pH 7.4) is given in Table 1. All the co-solvents were found to increase the dissolution rate of drug and the amount of drug dissolved increased with increase in the concentration of the co-solvent in each case. The dissolution rate was found to vary from 20 µg/ml/h in water to 125 µg/ml/h in the presence of co-solvents; the values were highest in ethanol followed by glycerol and PG. It is also seen that for each solvent, the dissolution rate improved considerably when water was replaced by PB (pH 7.4) for the preparation of co-solvent solutions.

**TABLE 1:** AMOUNT DISSOLVED AND INITIAL DISSOLUTION RATE FOR ENROFLOXACIN IN VARIOUS CO-SOLVENTS AT 25°

Solvent	Amount dissolved* (μg/ml)		Initial dissolution rate (μ/ml/hr)	
		
	Water	Buffer	Water	Buffer
Water	70.002	-	20.001	-
PB (7.4)	-	107.460	-	30.703
10% PEG 400	78.819	194.074	22.520	55.450
20% PEG 400	88.287	246.154	25.225	70.330
40% PEG 400	100.475	494.502	28.707	141.286
10% PG	107.012	135.105	30.575	38.602
20% PG	132.654	256.385	37.901	73.253
40% PG	325.991	1203.703	93.141	343.915
10% Glycerol	102.555	170.552	29.302	48.729
20% Glycerol	156.062	201.992	44.589	57.712
40% Glycerol	364.849	753.176	104.243	215.193
10% Ethanol	166.090	343.803	47.454	98.229
20% Ethanol	213.423	568.357	60.978	162.387
40% Ethanol	439.821	1300.383	125.663	371.358
25 mM CTAB**	300.184	612.907	85.767	175.116
50 mM CTAB**	458.186	930.737	130.991	265.925
25 mM SDS**	2517.373	2852.572	719.249	815.021
50 mM SDS**	3314.075	3478.288	946.878	993.796
25 mM Tween-80**	173.668	197.781	49.619	56.508
50 mM Tween-80**	312.696	320.678	89.342	91.622

* Amount dissolved at 3.5 h.

** Reported concentrations are micellar concentrations. Total surfactant concentration= micellar concentration+CMC. CMC values taken for CTAB, SDS and Tween-80 are 1 mM, 8 mM and 0.01 mM, respectively. PB=0.1M phosphate buffer (pH 7.4), PG = propylene glycol, PEG= polyethylene glycol, CTAB= cetyltrimethylammonium bromide, SDS= sodium dodecylsulphate.

Since the amount of drug dissolved was small at 10% co-solvent concentration, twenty four hour solubility data was obtained only for 20 and 40% concentration of co-solvent in water as well as phosphate buffer medium. Results are given in Table 2. In aqueous medium, the solubility was found to increase from about 146 µg/ml in water to about 439 µg/ml in the presence of co-solvents. Again highest solubility was obtained in ethanol followed by glycerol and PG. Although large solubility enhancement was observed, the maximum solubility was much less than l mg/ml.

**TABLE 2:** SOLUBILITY AND SOLUBILITY RATIOS FOR ENROFLOXACIN IN VARIOUS SOLVENTS AT 25°

Solvent	Solubility (μg/ml)*		Solubility Ratio			
		
	Water	Buffer	$\frac{S_{co-sol\left(w\right)}}{S_{w}}$	$\frac{S_{co-sol\left(b\right)}}{S_{b}}$	$\frac{S_{co-sol\left(b\right)}}{S_{w}}$	$\frac{S_{co-sol\left(b\right)}}{S_{co-sol\left(w\right)}}$
Water	145.868	-	-	-	-	-
PB (7.4)	-	181.916	-	-	-	1.247
20% PEG 400	162.009	300.175	1.111	1.650	2.058	1.854
40% PEG 400	188.799	555.725	1.294	3.055	3.810	2.943
20% PG	210.214	465.737	1.441	2.560	3.193	2.216
40% PG	399.349	1216.786	2.738	6.689	8.342	3.047
20% Glycerol	179.289	370.402	1.229	2.036	2.539	2.067
40% Glycerol	428.791	755.153	2.939	4.151	5.177	1.761
20% Ethanol	214.541	578.212	1.471	3.178	3.964	2.695
40% Ethanol	492.771	1699.342	3.378	9.341	11.650	3.448
25 mM CTAB**	324.813	723.902	2.227	3.979	4.963	2.229
50 mM CTAB**	426.783	1050.439	2.926	5.774	7.201	2.461
25 mM SDS**	2598.398	2914.401	21.730	16.020	19.979	1.122
50 mM SDS**	3385.296	3793.154	23.208	20.851	26.004	1.121
25 mM Tween-80**	217.547	232.969	1.491	1.281	1.597	1.07
50mM Tween-80**	342.670	351.786	2.349	1.934	2.412	1.027

* Time period for drug dissolution was kept 24 h for solubility data.

** Reported concentrations are micellar concentrations. Total surfactant concentration = Micellar concentration + CMC. CMC values taken for CTAB, SDS and Tween-80 are 1 mM, 8 mM and 0.01 mM, respectively. PB=0.1M phosphate buffer (pH 7.4), PG = propylene glycol, PEG = polyethylene glycol, CTAB= cetyltrimethylammonium bromide, SDS= sodium dodecylsulphate.

Since the solubility of enrofloxacin in PB (pH 7.4) was higher than that in water, it was thought of interest to study the combined effect of co-solvents and buffer. For this purpose solubility was determined in the presence of 20 and 40% co-solvent solutions prepared in PB (pH 7.4). Solubility in PB (pH 7.4) in the absence and presence of 20 and 40% co-solvent solutions is given in Table 2. The presence of co-solvent as well as buffer produced a very large increase in solubility in all cases.

Solubility enhancement has been expressed in terms of solubility ratios, S_co-sol(w)_/S_w_, S_co-sol(b)_/S_b_, S_co-sol(b)_/S_w_ and S_co-sol(b)/_S_co-sol(w)_, where S_co-sol(w)_ and S_w_ are the solubility of drug in the presence and absence of co-solvent, respectively and S_co-sol(b)_ and S_b_ are the corresponding values in buffer. Various solubility ratios in different solvents have also been recorded in Table 2. S_co-sol(w)_/S_w_ ratio, which represents the enhancement of aqueous solubility in the presence of co-solvent in water, was found to vary from 1.111 to 3.378. However, when the co-solvent solutions were prepared in buffer, the corresponding enhancement in buffer solubility (S_co-sol(b)_/S_b_ ratio) was 3.055 to 9.341 times. The total solubility enhancement due to the combined effect of co-solvent and buffer, expressed as solubility ratio S_co-sol(b)/_S_w_, was found to be in the range 3.810 to 11.650. Significant solubility enhancement could, therefore, be achieved. Enrofloxacin could be dissolved up to about 1.7 and 1.2 mg/ml in ethanol and PG, respectively corresponding to 11.65 and 8.33 times enhancement of aqueous solubility of enrofloxacin.

Higher pH of buffer can cause only a small increase in solubility since enrofloxacin is amphoteric[2]. The ratio of drug solubility in buffer and water (S_b/_S_w_) is only 1.247. For a given co-solvent, the ratio of solubility in buffer and water medium (S_co-sol(b)/_S_co-sol(w)_ ratio in Table 2), was found to be in the range 1.761 to 3.448 only. Thus large increase in solubility when the co-solvent solutions are prepared in buffer, cannot be due to increase in pH or dissolution of drug by buffer components. The presence of buffer and co-solvents has synergistic effect. The efficiency of the co-solvents increases in the presence of buffer components. This result is important especially since the buffer used is biocompatible and at near neutral pH. The only explanation which can be offered for the dramatic increase in the solubility when co-solvent solutions are prepared in buffer is given below.

The solubilization efficiency of a solvent is a function of the relative magnitudes of the various solute-solute, solute-solvent and solvent-solvent interactions. Reduction in solute-solute and solvent-solvent interactions should increase the solubilization efficiency. The co-solvents used in the present work have a tendency to form inter- and intra-molecular hydrogen bonds. Higher pH of buffer and increase in the ionic strength due to the presence of buffer components should decrease the ability of co-solvent molecules to form inter- and intra-molecular hydrogen bonds resulting in decrease in solvent-solvent interactions and consequent increase in solubility.

For a given solvent system, the solubilization power gives a quantitative estimate of the solubilization potential of the co-solvent[7]. The solubilization power (Φ) of various co-solvents was determined using equation, log S_mix_= log S+ΦV_co-sol_, where S_mix_ and S are the solubilities of drug in solvent mixture and pure solvent, respectively. V_co-sol_ is the volume fraction of the co-solvent and Φ is defined as the solubilization power of the co-solvent. The Φ values for various solvents are given in Table 3. The order of solubilization potential was found to vary as ethanol>glycerol>PG>PEG 400 in water and ethanol>PG>glycerol>PEG 400 in buffer. Dielectric constants of the solvents show that the polarity of the solvent varies as glycerol>PG>ethanol>PEG 400 (Table 3). It appears that the polarity of co-solvent is not the only factor affecting solubility of drug. PEG 400 is not a good solvent for enrofloxacin in spite of it being least polar. It appears that the ability of solvent to form hydrogen bonds with the hetero-atoms in the drug molecule is another important factor governing the solubility of drug. Similar findings have also been reported by Seedher and Bhatia[8]. Since the drug exists in zwitterionic form at neutral pH, hydrophobic interactions with the non-polar part of PEG are also not significant.

**TABLE 3:** SOLUBILIZATION POWER FOR VARIOUS CO-SOLVENTS IN WATER AND BUFFER

Solvent	Dielectric constant of co-solvent	Solubilization power (φ)	
		
		Water	Buffer
PEG 400	12.4	0.2801	1.2125
Ethanol	24.3	1.3218	2.4263
PG	32.0	1.0938	2.0635
Glycerol	42.5	1.1707	1.5458

φ values were obtained using equation log S_mix_= log S+ΦV_co-sol_, where S_mix_ and S are the solubilities of drug in solvent mixture and pure solvent, respectively. PB=0.1M phosphate buffer (pH7.4), PG = propylene glycol, PEG = polyethylene glycol.

Since the solubility enhancement using PEG as co-solvent was least, the use of 1:1 and 2:1 PEG 8000:drug solid dispersions was also tried. Due to the formation of a sticky mass, which was difficult to dry, solid dispersions could not be prepared using PEG 400. The solubility of solid dispersions in water and PB (pH 7.4) was found to be 233 and 486 μg/ml, respectively. Thus aqueous solubility increased from 146 to 233 μg/ml, about 1.6 times. The total increase in aqueous solubility due to the combined effect of solid dispersion and buffer (146 to 486 μg/ml) was 3.33 times.

Surfactants are known to solubilize poorly-soluble drugs at concentrations above CMC[9–10]. Solubility of enrofloxacin in CTAB, SDS and Tween 80 at 25 and 50 mM micellar concentration in water and PB (pH 7.4) is given in Table 2. Tween 80 was not found to be a good solvent for enrofloxacin. Amongst ionic surfactants, SDS was found to be a much better solvent as compared to the cationic surfactant, CTAB. Solubility was found to be exceptionally high in SDS. About 3.4 and 3.8 mg/ml of enrofloxacin could be dissolved in water and buffer medium, respectively at 50 mM micellar concentration of SDS.

A very high solubility of drug in SDS shows that the non-polar part of the molecule is solubilized into the micellar interior while positively charged piperazinyl groups are in the outer core, decreasing the repulsive forces between the head groups of the surfactant molecules, thereby decreasing CMC, increasing aggregation number and volume of micelles and increasing solubilization. Much lower solubility in CTAB showed that the orientation of solubilized molecules is such that the negatively charged carboxyl groups do not take part in solubilization. Tween 80 also enhanced the solubility of drug but the solubilization power was lower than ionic surfactants. In non-ionic surfactant, the drug appears to be located preferentially in the palisade layer and is stabilized by formation of hydrogen bonds with polyoxyethylene groups in the surfactant. This is possible since the drug molecule contains seven hydrogen bond acceptors and one hydrogen bond donor.

Thermodynamically the solubilization can be considered as partitioning of the drug between micellar and aqueous phase, and the standard free energy of solubilization ($\Delta G_{S}^{0}$) can be represented by the expression $\Delta G_{S}^{0}$ = -RT ln K, where R is the gas constant, T is the absolute temperature and K is the partition coefficient of drug between micellar and aqueous phase[11]. The micelle-water partition coefficient (K) is the ratio of the drug concentration in the micelle to the drug concentration in water for a particular surfactant concentration and is given by K= (S_tot_-S_w_)/S_w_. K and $\Delta G_{S}^{0}$ values for various surfactants are given in Table 4. Surfactant solubilization parameters are found to be very low for Tween 80 and exceptionally high for SDS and thus SDS is an excellent solvent for enrofloxacin.

**TABLE 4:** SOLUBILIZATION PARAMETERS FOR VARIOUS SURFACTANTS

Solvent*	Surfactant Solubilization parameters			
	
	K		$\Delta \;G_{S}^{0}$ (kJ mol^−1^)	
		
	Water	Buffer	Water	Buffer
25 mM CTAB	1.227	3.963	−0.507	−3.412
50 mM CTAB	1926	6.201	−1.624	−4.522
25 mM SDS	16.813	18.979	−6.993	−7.294
50 mM SDS	22.208	25.004	−7.683	−7.977
25 mM Tween-80	0.491	0.597	+1.762	+1.278
50 mM Tween-80	1.349	1.412	−0.742	−0.855

* Reported concentrations are micellar concentrations. Total surfactant concentration = Micellar concentration + CMC. CMC values taken for CTAB, SDS and Tween-80 are 1 mM, 8 mM and 0.01 mM, respectively. $\Delta \;G_{S}^{0}$ is the standard free energy of solubilization of drug and K is the partition coefficient of drug between micellar and aqueous phase. CTAB= cetyltrimethylammonium bromide, SDS= sodium dodecylsulphate, Tween-80 = polyoxyethylene (20) sorbitan monooleate.

Since the commonly used dose of enrofloxacin is 5.7 mg, the minimum solubility of drug required for 10 ml, 5 ml and 2 ml doses are 570, 1140 and 2850 µg/ml, respectively. Solvents with drug solubility greater than that required for 10 ml, 5 ml and 2 ml doses are given in Table 5. The data can be useful for the development of parenteral formulations of this drug.

**TABLE 5:** SOLVENTS WITH DRUG SOLUBILITY GREATER THAN THAT REQUIRED FOR MINIMUM DOSE

Solvents with drug solubility greater than that required for		

10 ml dose	5 ml dose	2 ml dose
40% glycerol in buffer,	40% PG in buffer,	25 mM SDS in buffer,
20% ethanol in buffer,	40% ethanol in buffer,	50 mM SDS in water and buffer
25 & 50 mM CTAB in buffer,	25 mM SDS in buffer,	
40% PG in buffer,	50 mM SDS in water and buffer	
40% ethanol in buffer,		
25 mM SDS in buffer,		
50 mM SDS in water and buffer		

Dose = 5.7 mg, Required drug solubility for: 10 ml dose = 570 μg/ml, 5 ml dose = 1140 μg/ml, 2 ml dose = 2850 μg/ml.
